# Safety profile of azvudine in COVID-19 patients with renal impairment: a retrospective analysis

**DOI:** 10.3389/fphar.2026.1830085

**Published:** 2026-06-29

**Authors:** Xiaomin Zhang, Jiajian Qian, Jiawen Cui, Yuhong Luo, Sichao Huang, Zhiyong Wen

**Affiliations:** 1 Department of Pharmacy, The Fifth Affiliated Hospital of Zunyi Medical, University, Zhuhai (Zhuhai Sixth People’s Hospital), Zhuhai, China; 2 Department of Pharmacy, Zhuhai People’s Hospital (The Affiliated Hospital of Beijing Institute of Technology, Zhuhai Clinical Medical College of Jinan University), Zhuhai, China; 3 Department of Pharmacy, Guangzhou Twelfth People’s Hospital, Guangzhou, China

**Keywords:** azvudine, nomogram model, renal impairment, retrospective analysis, safety

## Abstract

**Objective:**

Azvudine (FNC), an antiviral drug approved in China for the treatment of Coronavirus disease 2019 (COVID-19) infection, has not yet established a well-defined safety profile in patients with renal impairment. This study aimed to evaluate the safety of using FNC in patients with renal impairment.

**Methods:**

We conducted a retrospective analysis of clinical data from 328 COVID-19 patients to assess FNC-related adverse drug events (ADEs). Patients were stratified into two cohorts: a control group with an estimated glomerular filtration rate (eGFR) > 70 mL/min/1.73 m^2^ and a study group with eGFR ≤70 mL/min/1.73 m^2^. Our main objective was to assess whether there were any differences in the incidence of ADEs between the two groups. Secondly, we attempted to develop predictive models for ADEs that were frequent and closely related to the eGFR grade.

**Results:**

Over half of the cohort (51.8%) experienced FNC-associated ADEs, predominantly hepatic function abnormalities (36.6%) and renal function abnormalities (18.9%). The incidence of acute kidney injury (AKI) was 8.8%. Patients with renal impairment exhibited a higher risk of ADEs, particularly among elderly individuals and patients with a high neutrophil count. No differences in hepatic function abnormalities were found between the two groups. Patients with renal impairment had a higher risk of renal function abnormalities and AKI, with decreased eGFR identified as an independent risk factor for these ADEs. The predictors included in the nomogram model for AKI were neutrophil count and eGFR grade. The nomogram model demonstrated predictive performance and calibration, with an area under the curve of 0.812.

**Conclusion:**

These findings highlight the need for enhanced safety monitoring and potential dose adjustments when administering FNC to COVID-19 patients with renal impairment.

## 1 Introduction

Azvudine (FNC) is a novel dual-target inhibitor that acts on both nucleoside reverse transcriptase and the HIV-1 auxiliary protein Vif, demonstrating significant antiviral activity (Wang et al., 2014; Chang, 2022; Fayzullina et al., 2022). FNC inhibits the replication of positive-sense RNA viruses by targeting RNA-dependent polymerases, including viruses such as HCV, HBV, SARS-CoV-2, retroviruses like HIV, and human endogenous retroviruses (HERVs) (Fayzullina et al., 2022). The antiviral mechanism of FNC primarily involves a three-step phosphorylation process mediated by host kinases, converting FNC into its active form. Once activated, FNC is incorporated into viral RNA during replication, where it blocks RNA strand elongation, thereby halting viral RNA synthesis and replication (Yu and Chang, 2020; Zhang et al., 2021). Clinical studies have demonstrated that FNC is a long-acting oral antiviral agent, with a half-life exceeding 120 h in target cells (Sun et al., 2020). Randomized controlled trials had shown that FNC significantly reduces the time to nucleic acid conversion and improves clinical outcomes in patients infected with SARS-CoV-2 (Chen and Tian, 2023).

The China National Medical Products Administration (NMPA) has approved FNC for treating mild-to-moderate SARS-CoV-2 infection in adults (Zhang et al., 2023), but advises caution in patients with moderate-to-severe renal impairment due to its predominant renal clearance pathway. Emerging pharmacoepidemiologic studies reveal that adverse drug events (ADEs) occur with higher frequency and greater severity in chronic kidney disease (CKD) patients (Datta et al., 2024). Notably, renal dysfunction is a prevalent comorbidity among Coronavirus disease 2019 (COVID-19) patients. SARS-CoV-2 infection is associated with increased renal compromise, where patients with CKD exhibit significantly elevated mortality (9.4% vs. 5.8%) and hospitalization rates (41.5% vs. 28.5%) compared to those with preserved renal function (Pakhchanian et al., 2021). Nevertheless, the prescribing information of FNC indicated that previous clinical trials excluded patients with an estimated glomerular filtration rate (eGFR) < 70 mL/min/1.73 m^2^. Therefore, the instructions suggested that FNC should be used cautiously in patients with moderate to severe renal impairment. Due to the metabolic characteristics, FNC may exhibit altered pharmacokinetic profiles and increased systemic accumulation in individuals with renal impairment, elevating the likelihood of dose-dependent toxicities. Detailed dosage recommendations for patients with renal insufficiency are available in the prescribing information of other COVID-19 treatments including remdesivir, molnupiravir and nirmatrelvir/ritonavir. Azvudine is priced lower than the above three drugs in China, making it clinically valuable to explore its safety profile in renally impaired patients.

Consequently, the primary objective of this study was to evaluate the safety profile of FNC in COVID-19 patients with renal impairment to inform optimized therapeutic dosing. Specifically, we employed a cohort of patients with normal renal function who received FNC as a control group to compare the incidence of total and specific adverse events against those observed in patients with renal injury. Secondly, based on the collected data, this study attempted to establish a model for predicting adverse drug events strongly associated with renal insufficiency, thereby providing practical references for clinical diagnosis and treatment.

## 2 Methods

### 2.1 Design, setting, and participants

We retrospectively reviewed medical records of SARS-CoV-2-infected inpatients treated with FNC between 1 December 2022, and June 1, 2025 at a tertiary hospital in Zhuhai, China (Zhuhai People’s Hospital Medical Group). The inclusion criteria were: (1) age ≥18 years; (2) treatment duration of FNC ≥2 consecutive days or discontinuation after 1 day due to drug-related adverse events; (3) available data of complete blood count, hepatic function and renal function pre- and post- FNC treatment. The exclusion criteria were as follows: (1) diagnosis of active malignancy; (2) acute exacerbation of neurological disorders; (3) decompensated liver cirrhosis; (4) pregnancy or lactation. This study was reviewed and approved by the Ethics Committee of Zhuhai People’s Hospital (Approval No (2024) Ethical Review [Research] No. 113). As this study was a retrospective analysis, the committee waived the requirement for informed consent from individual patients.

Demographic and clinical parameters were documented, including age, sex, body weight, FNC dosage and duration, concomitant medications, hepatic function, renal function, coagulation profiles, complete blood counts, C-reactive protein (CRP), procalcitonin (PCT) and ADEs. According to approved prescribing guidelines, patients were stratified into two cohorts: a control group with estimated glomerular filtration rate (eGFR) > 70 mL/min/1.73 m^2^ and a study group with eGFR ≤70 mL/min/1.73 m^2^ eGFR was calculated using the CKD-EPI (Chronic Kidney Disease Epidemiology Collaboration) formula. Comparative analysis of ADE incidence was conducted between the two cohorts.

### 2.2 Definitions of ADEs

FNC-associated ADEs were classified using the Chinese Hospital Pharmacovigilance System into five causality categories: definite, probable, possible, unlikely, and unassessable. ADEs categorized as “definite”, “probable” or “possible” were attributed to FNC. The Chinese hospital pharmacovigilance system determined the causal relationship of ADEs according to the standards of the WHO Uppsala Monitoring Centre (UMC). Consensus assessments were independently performed by five clinical pharmacists. When the evaluations of 5 clinical pharmacists were inconsistent, the causal relationship between the drug and the adverse event was judged according to the rule of the minority obeying the majority. Categories of ADEs included: drug-associated hepatic parameter abnormalities, drug-induced liver injury, cholestatic hepatitis, grading of transaminase elevation, grading of γ-glutamyl transpeptidase elevation, drug-associated renal function abnormalities, acute kidney injury, grading of creatinine elevation, diarrhea, nausea/vomiting, hematologic adverse events, hypersensitivity reactions, and neurologic toxicities. The above ADEs were defined with reference to relevant guidelines or the literature and were provided in Supplementary Tables S1,S2 (Brennan et al., 2021; European Association for the Study of the Liver, 2009; Kellum et al., 2013; Tamma et al., 2017).

Hepatic function indicators include: alanine transaminase (ALT), aspartate aminotransferase (AST), alkaline phosphatase (ALP), γ-glutamyl transpeptidase (GGT), total bilirubin (TBIL), direct bilirubin (DBIL), and indirect bilirubin (IBIL). Renal function indicators included: serum creatinine (Crea) or blood urea nitrogen (Urea). We set the degree of elevation of the transaminase and GGT as 0, 1, 2 and 3, respectively. We also set the degree of elevation of Crea as 0, 1, 2, 3 and 4. A higher numerical value represents a more pronounced elevation.

### 2.3 Outcome measures

The primary outcome was the comparative incidence rate of total ADEs between the study and control groups, calculated as (number of patients with ≥1 ADE per group/total patients in the group) × 100%. Secondary outcomes were incidence rates of individual ADEs in the two groups (number of occurrences per ADE/total patients in the group) × 100%.

According to renal function levels, patients were categorized into four subgroups based on eGFR: (1) Grade 1: eGFR 45–70 mL/min/1.73 m^2^; (2) Grade 2: eGFR 30–45 mL/min/1.73 m^2^; (3) Grade 3: eGFR 15–30 mL/min/1.73 m^2^; (4) Grade 4: eGFR <15 mL/min/1.73 m^2^.

### 2.4 Statistical analysis

Statistical analyses were performed using SPSS version 25.0. Categorical variables were presented as percentages and compared using the Chi-square test or Fisher’s exact test. Quantitative variables with normal distribution were expressed as mean ± standard deviation (SD) and analyzed via Student’s t-test or one-way ANOVA when they possessed equal variances. Non-normally distributed quantitative variables were reported as median (interquartile range, IQR), and differences between groups were assessed using the Mann-Whitney U test. A two-tailed P < 0.05 was considered statistically significant. We also conducted subgroup analyses on eGFR and age to examine the impact of these two confounding factors on ADEs. For the analysis of abnormal liver function, we also analyzed the abnormal elevation of transaminases and GGT as a sensitivity analysis. For the analysis of abnormal renal function, we also analyzed the elevated serum creatinine and the incidence of AKI as a sensitivity analysis. Moreover, to reduce potential bias caused by inconsistent evaluation criteria among clinical pharmacists when judging FNC-associated ADEs, we performed logistic regression on ADEs with high incidence. If the missing data of the observation endpoint or covariate was less than 5% of the total data volume, simple random imputation, mean or median imputation was adopted. If the amount of missing data was more than 5% of the total data volume, the deletion method was adopted.

We attempted to develop predictive models for ADEs that were frequent and closely related to the eGFR grade. Variables with P < 0.05 in univariate analysis were incorporated into multivariate logistic regression models after excluding collinear parameters. Risk factors derived from multivariate logistic regression were regarded as potential predictive factors. We further adopted the least absolute shrinkage and selection operator (LASSO) regression with 10-fold cross-validation to screen optimal predictive variables based on the minimum criterion within one standard error. The Wald chi-square test was employed to select predictors for constructing a nomogram to predict the occurrence of ADEs in patients using FNC. The receiver operating characteristic (ROC) curve was used to calculate the area under the curve (AUC) to assess the discriminatory ability of the model. Next, calibration curves were plotted to determine whether the predicted and observed probabilities for the occurrence of ADEs were in concordance. Bootstrap resampling (500 resamples) was used for this plot. To assess whether there was multicollinearity among the independent variables included in the predictive model, we used the Variance Inflation Factor (VIF) for testing. When VIF ≥10, it indicated that there was severe multicollinearity among the independent variables. Assume that data missing was completely random missing.

## 3 Results

### 3.1 Patient characteristics

**FIGURE 1:**
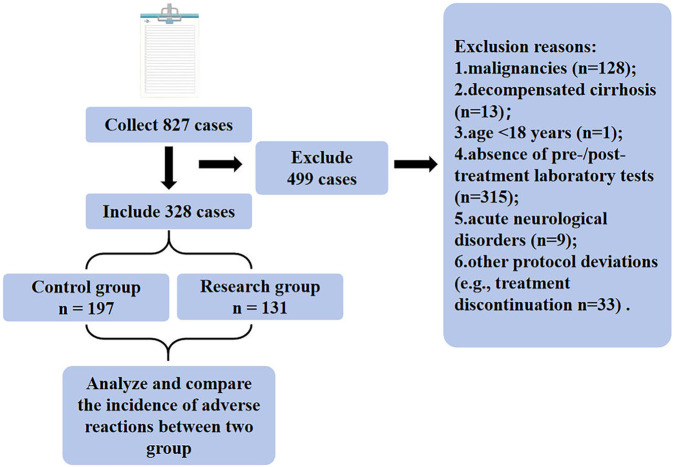
Flow chat showing the research process.

A total of 827 medical records were initially reviewed, with 328 patients meeting the inclusion criteria after excluding 499 cases. The cohort comprised 196 control group patients (eGFR >70 mL/min/1.73 m^2^) and 132 study group patients (eGFR ≤70 mL/min/1.73 m^2^) (Figure 1). Demographic analysis revealed a male predominance (66.8%), with the majority aged >60 years (60–80 years: 42.7%; >80 years: 44.2%). Over half of the cohort (51.8%) experienced FNC-associated ADEs, predominantly hepatic function abnormalities (36.6%) and renal function abnormalities (18.9%) (Table 1). The most common type of liver ADEs was transaminase elevation (32.6%). Renal function abnormalities primarily presented as BUN elevation (17.1%).

**TABLE 1:** Baseline characteristics.

Characteristic	n (%, 95% CI)
Include patients	328
Gender	-
Male	219
Female	109
Age (years)	-
<45	12
45–60	31
60–80	140
≥80	145
BMI	-
<18.5	32
18.5–24	160
24–28	106
>28	30
eGFR (mL/min/1.73 m^2^)	-
>70	196
45–70	78
30–45	23
15–30	10
0–15	21
The severity of COVID-19	-
Mild to moderate infections	194
Severe/Critical infection	134
ADEs	170 (51.8, 46.4–57.2)
Drug-associated hepatic parameter abnormalities	121 (36.6, 32.6–41.9)
Transaminase elevation	107 (32.6 27.8–37.9)
GGT elevation	63 (19.2 15.3–23.8)
ALP elevation	21 (6.4, 4.2–9.6)
TBIL elevation	14 (4.3, 2.6–7.0)
Drug-induced liver injury	10 (3.1, 1.7–5.5)
Drug-induced cholestatic hepatitis	3 (0.91, 0.31–2.7)
Drug-associated renal function abnormalities	62 (18.9, 15.0–23.5)
Urea elevation	56 (17.1, 13.4–21.5)
Serum creatinine elevation	37 (11.3 8.3–15.2)
Acute kidney injury	29 (8.8, 6.2–12.4)
Decreased blood potassium	25 (7.6 5.2–11.0)
Decreased blood calcium	24 (7.3, 5.0–10.7)
Blood creatine kinase elevation	18 (5.5, 3.5–8.5)
Decreased platelet count	12 (3.7, 2.1–6.3)
Gastrointestinal discomfort	3 (0.91, 0.31–2.7)

BMI: body mass index; GGT:γ-glutamyl transpeptidase; ALP:alkaline phosphatase; TBIL:total bilirubin; Urea: Urea nitrogen; ADEs:.Adverse drug events; 95% CI: 95% confidence interval.

### 3.2 Analysis of overall ADEs

**FIGURE 2:**
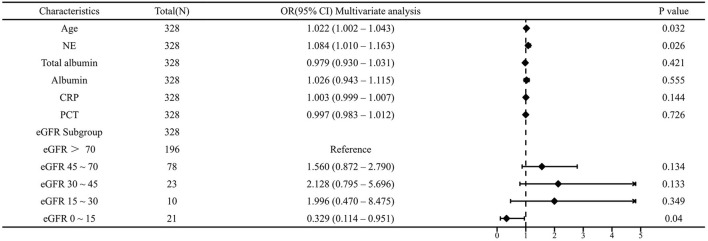
Forest plot of logistic regression analysis of overall ADEs.

The incidence of ADEs was significantly higher in the study group compared to the control group (P = 0.031). Patients with elevated baseline laboratory tests including white blood cell count (WBC), neutrophil count (NE), lymphocyte count (LY), CRP and PCT or with hypoalbuminemia, demonstrated heightened susceptibility to ADEs. Intriguingly, FNC dosage, treatment duration, and cumulative dose showed no significant association with ADEs. Subgroup analyses revealed increased incidence of ADEs among patients with eGFR 45–70 mL/min/1.73 m^2^ and 30–45 mL/min/1.73 m^2^ (Table 2). Age and baseline neutrophil count emerged as independent risk factors for ADEs (P < 0.05), suggesting that advancing age and elevated neutrophil levels may be correlated with amplified ADE risks (Figure 2).

**TABLE 2:** Analysis of overall ADEs.

Project	ADEs	No-ADEs	P value
Control group (n, %)	92 (54.1)	104 (65.8)	-
Research group (n, %)	78 (45.9)	54 (34.2)	0.031
Subgroup (mL/min/1.73 m^2^)	-	-	-
eGFR 45–70 (n, %)	49 (34.8)	29 (21.8)	0.018^3^
eGFR 30–45 (n, %)	16 (14.8)	7 (6.3)	0.040^3^
eGFR 15–30 (n, %)	7 (7.1)	3 (2.8)	0.20^3^
eGFR 0–15 (n, %)	6 (6.1)	15 (12.6)	0.11^3^
Dialysis treatment (n, %)	6 (3.5)	10 (6.3)	0.24^1^
Gender (male, n, %)	117 (68.8)	102 (64.6)	0.41
Ages (Year)^1^	80 (70, 85)	77 (65, 83)	0.005
BMI^2^	23.2 ± 3.7	23.7 ± 3.7	0.19
Laboratory examination before medication	-	-	-
WBC (×10^9^/L)^1^	7.5 (4.8, 10.7)	6.2 (4.8, 8.0)	0.010
NE (×10^9^/L)^1^	5.7 (3.5, 9.3)	4.5 (3.3, 6.5)	0.004
LY (×10^9^/L)^1^	0.74 (0.50, 1.1)	0.92 (0.63, 1.3)	0.002
Hb (g/L)^1^	123.0 (106.0, 138.2)	124.0 (107.7, 138.2)	0.76
PLT (×10^12^/L)^1^	180.0 (129.0, 231.2)	173.5 (127.7, 236.2)	0.98
Total protein (g/L)^2^	59.2 ± 8.1	60.9 ± 8.0	0.054
ALB (g/L)^2^	33.3 ± 5.3	34.6 ± 5.3	0.028
CRP (mg/L)^1^	72.4 (22.3, 112.7)	53.1 (19.2, 87.9)	0.020
PCT (ng/ml)^1^	0.11 (0.05, 0.85)	0.05 (0.05, 0.31)	0.006
The daily dosage of FNC (mg)^1^	5 (2, 5)	5 (2, 5)	0.70
FNC treatment course (d)^1^	7 (1, 17)	7 (2, 14)	0.15
The cumulative dose of FNC (mg)^1^	35 (5, 85)	35 (9, 70)	0.28

1: median, IQR; 2: mean ± SD; 3: Compared with the control group; -: no data; BMI: body mass index; WBC: white blood cell count; NE: neutrophil count; LY: lymphocyte count; Hb: Hemoglobin; PLT: platelet; ALB: albumin; CRP: C-reactive protein; PCT: procalcitonin; FNC: azvudine.

### 3.3 Hepatic adverse events

**FIGURE 3:**
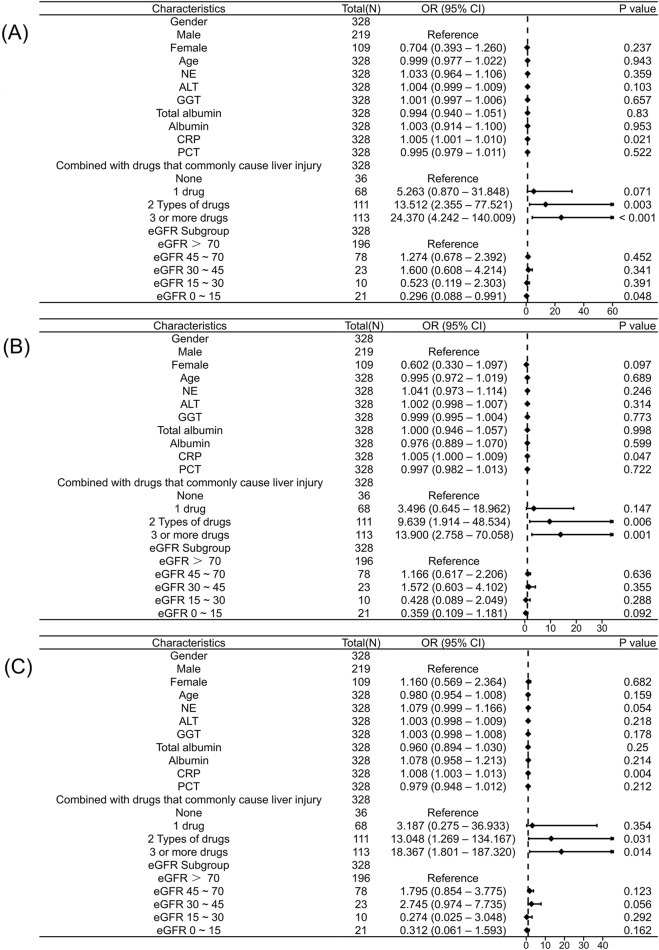
Logistics regression forest plot of hepatic adverse events. **(A)** Forest plot of logistic regression analysis of hepatic parameter abnormalities. **(B)** Forest plot of logistic regression analysis of transaminase elevation. **(C)** Forest plot of logistic regression analysis of GGT elevation.

The incidence rates of hepatic adverse events in the study group were similar to those in the control group, which included hepatic function abnormalities, transaminase elevation, and GGT elevation (P > 0.05). However, subgroup analyses revealed significantly elevated incidence rates of hepatic adverse events in patients with eGFR 30–45 mL/min/1.73 m^2^ compared to controls (P < 0.05) (Table 3). FNC dosing regimens including daily dose, cumulative dose and duration may not be associated with hepatic adverse events (Supplementary Table S3). Patients with elevated baseline levels of WBC, neutrophil count, ALT, AST, CRP, or PCT, or reduced total protein prior to FNC initiation demonstrated a significantly higher incidence of hepatic adverse events post-treatment (P < 0.05). A significant relationship was observed between the number of concomitant medications commonly causing hepatic function abnormalitie and the incidence of hepatic adverse events. Notably, co-administration with cephalosporins, meropenem, baricitinib, or nadroparin (low molecular weight heparin) was associated with a disproportionately higher risk of hepatic injury (Table 4). No significant differences were observed between the study and control groups in the incidence of acute drug-associated hepatitis or drug-related cholestatic hepatitis. Additionally, univariate analysis indicated that male gender, hypoalbuminemia or combined statin use were associated with higher risk of elevated transaminases. Moreover, patients with eGFR 30–45 mL/min/1.73 m^2^ demonstrated significantly higher rates of grade 2 transaminase elevation (P = 0.044) and grade 1 GG T elevation (P = 0.002) compared to the control group (Supplementary Table S5, S6). Logistic analysis showed that the co-administration of two or more drugs commonly causing hepatic function abnormalities and elevated CRP levels were independent risk factors (Figure 3A–C).

**TABLE 3:** Analysis of hepatic adverse events.

Project	Hepatic parameter			Transaminase elevation			GGT elevation		
	Abnormal	Normal	P value	Abnormal	Normal	P value	Abnormal	Normal	P value
Control group (n, %)	65 (53.7)	131 (63.3)	-	58 (54.2)	138 (62.4)	-	31 (49.2)	165 (62.3)	-
Research group (n, %)	56 (46.3)	76 (36.7)	0.088	49 (45.8)	83 (37.6)	0.15	32 (50.8)	100 (37.7)	0.057
Subgroup (mL/min/1.73 m^2^)	-	-	-	-	-	-	-	-	-
eGFR 45–70 (n, %)	34 (34.3)	44 (25.1)	0.11^3^	29 (33.3)	49 (26.2)	0.221^3^	20 (39.2)	58 (26.0)	0.059^3^
eGFR 30–45 (n, %)	13 (16.7)	10 (7.1)	0.027^3^	12 (17.1)	11 (7.4)	0.0281^3^	9 (22.5)	14 (7.8)	0.018^3^
eGFR 15–30 (n, %)	4 (5.8)	6 (4.4)	0.73^3^	3 (4.9)	7 (4.8)	1.01^3^	1 (3.1)	9 (5.2)	1.0^3^
eGFR 0–15 (n, %)	5 (7.1)	16 (10.9)	0.38^3^	3 (4.3)	16 (10.9)	0.111^3^	2 (6.1)	19 (10.3)	0.75^3^
Dialysis treatment (n, %)	5 (4.1)	11 (5.3)	0.63^3^	5 (4.7)	11 (5.0)	0.90^3^	2 (3.2) 1	14 (5.3) 1	0.75^3^
Gender (male, n, %)	93 (76.9)	126 (60.9)	0.003	84 (78.5)	135 (61.1)	0.002	46 (73.0)	173 (65.3)	0.24
Ages (Year)^1^	79 (70, 85)	77 (66, 83)	0.04	79.0 (70.0, 85.0)	77.0 (66.0, 84.0)	0.092	76.0 (68.0, 84.0)	78.0 (68.0, 84.0)	0.86
BMI^2^	22.9 ± 3.6	23.8 ± 3.7	0.032	23.1 (3.5)	23.6 (3.7)	0.20	23.8 (3.4)	23.4 (3.7)	0.42
Indicators before medication	-	-	-	-	-	-	-	-	-
WBC (×10^9^/L)^1^	7.9 (5.3, 11.1)	6.2 (4.7, 8.5)	0.001	7.9 (5.2, 11.1)	6.2 (4.8, 8.6)	0.003	8.8 (6.1, 11.6)	6.2 (4.7, 8.8)	<0.001
NE (×10^9^/L)^1^	6.5 (3.8, 9.6)	4.5 (3.3, 6.6)	<0.001	6.7 (3.6, 9.7)	4.5 (3.3, 6.7)	0.001	7.2 (4.7, 9.7)	4.6 (3.3, 7.2)	<0.001
ALT (U/L)^1^	25 (18.5, 42.0)	21 (14.0, 30.0)	<0.001	26.0 (19.0, 42.0)	21.0 (15.0, 30.0)	<0.001	26.0 (20.0, 41.3)	21.0 (15.0, 33.0)	0.003
AST (U/L)^1^	39.0 (26.0, 60.0)	26.7 (20.0, 37.0)	<0.001	41.0 (26.0, 60.0)	27.0 (20.0, 38.0)	<0.001	39.0 (28.0, 60.0)	27.0 (20.1, 42.0)	<0.001
GGT (U/L)^1^	37.0 (24.0, 62.5)	27.0 (19.0, 55.0)	0.005	36.0 (24.0, 57.0)	29.0 (19.0, 57.0)	0.062	52.0 (35.0, 72.0)	27.0 (19.0, 52.5)	<0.001
Total protein (g/L)^2^	58.5 ± 7.7	60.9 ± 8.2	0.008	58.2 ± 7.9	60.8 ± 8.1	0.006	58.7 ± 8.6	60.3 ± 8.0	0.14
ALB (g/L)^2^	32.7 ± 5.2	34.6 ± 5.3	0.002	32.5 ± 5.4	34.6 ± 5.2	<0.001	33.0 ± 5.3	34.1 ± 5.3	0.14
CRP (mg/L)^1^	83.2 (36.1, 130.7)	48.6 (17.2, 86.1)	<0.001	85.1 (36.5, 130.0)	48.6 (17.3, 87.7)	<0.001	88.9 (40.9, 148.1)	56.4 (19.0, 93.7)	<0.001
PCT (ng/ml)^1^	0.14 (0.05, 0.88)	0.05 (0.05, 0.38)	<0.001	0.14 (0.05, 0.94)	0.05 (0.05, 0.44)	0.001	0.14 (0.05, 1.1)	0.05 (0.05, 0.52)	0.005

1: median, IQR; 2: mean ± SD; 3: Compared with the control group; -: no data; BMI: body mass index; WBC: white blood cell count; NE: neutrophil count; ALT: alanine transaminase; AST: aspartate aminotransferase; GGT: γ-glutamyl transpeptidase; ALB: albumin; CRP: C-reactive protein; PCT: procalcitonin.

**TABLE 4:** The influence of combined medication on liver function.

Project	Hepatic parameter			Transaminase elevation			GGT elevation		
	Abnormal	Normal	P value	Abnormal	Normal	P value	Abnormal	Normal	P value
Combined drugs with the commonly incidence of abnormal liver function (n, %)	120 (99.2)	173 (83.6)	<0.001	106 (99.1)	187 (84.6)	<0.001	62 (98.4)	231 (87.2)	0.009
Combined types of drugs with the commonly incidence of abnormal liver function	-	-	-	-	-	-	-	-	-
1 drug (n, %)	13 (86.7)	55 (61.8)	0.061	11 (84.6)	57 (62.6)	0.21	5 (83.3)	63 (64.3)	0.66
2 drugs (n, %)	42 (95.5)	69 (67.0)	<0.001	39 (95.1)	72 (67.9)	<0.001	23 (95.8)	88 (71.5)	0.011
3 or more drugs (n, %)	64 (97.0)	49 (59.0)	<0.001	55 (96.5)	58 (63.0)	<0.001	34 (97.1)	79 (69.3)	<0.001
Combined the use of statins (n, %)	52 (43.0)	59 (28.5)	0.008	45 (42.1)	66 (29.9)	0.029	26 (41.3)	85 (32.1)	0.17
Combined the use of cephalosporin drugs for injection (n, %)	78 (64.5)	98 (47.3)	0.003	67 (62.6)	109 (49.3)	0.024	43 (68.3)	133 (50.2)	0.010
Combined the use of conazole antifungal drugs (n, %)	18 (14.9)	18 (8.7)	0.084	13 (12.1)	23 (10.4)	0.64	10 (15.9)	26 (9.8)	0.17
Combined the use of moxifloxacin (n, %)	22 (18.2)	29 (14.0)	0.31	21 (19.6)	30 (13.6)	0.16	11 (17.5)	40 (15.1)	0.64
Combined the use of meropenem (n, %)	13 (10.7)	6 (2.9)	0.003	12 (11.2)	7 (3.2)	0.003	8 (12.7)	11 (4.2)	0.015
Combined the use of baricitinib (n, %)	12 (9.9)	3 (1.4)	<0.001	11 (10.3)	4 (1.8)	0.001	9 (14.3)	6 (2.3)	<0.001
Combined the use of low molecular weight heparin (n, %)	87 (71.9)	85 (41.1)	<0.001	75 (70.1)	97 (43.9)	<0.001	47 (74.6)	125 (47.2)	<0.001
Combined the use of rivaroxaban (n, %)	7 (5.8)	13 (6.3)	0.86	6 (5.6)	14 (6.3)	0.80	6 (9.5)	14 (5.3)	0.24

no data.

### 3.4 Renal adverse events

**FIGURE 4:**
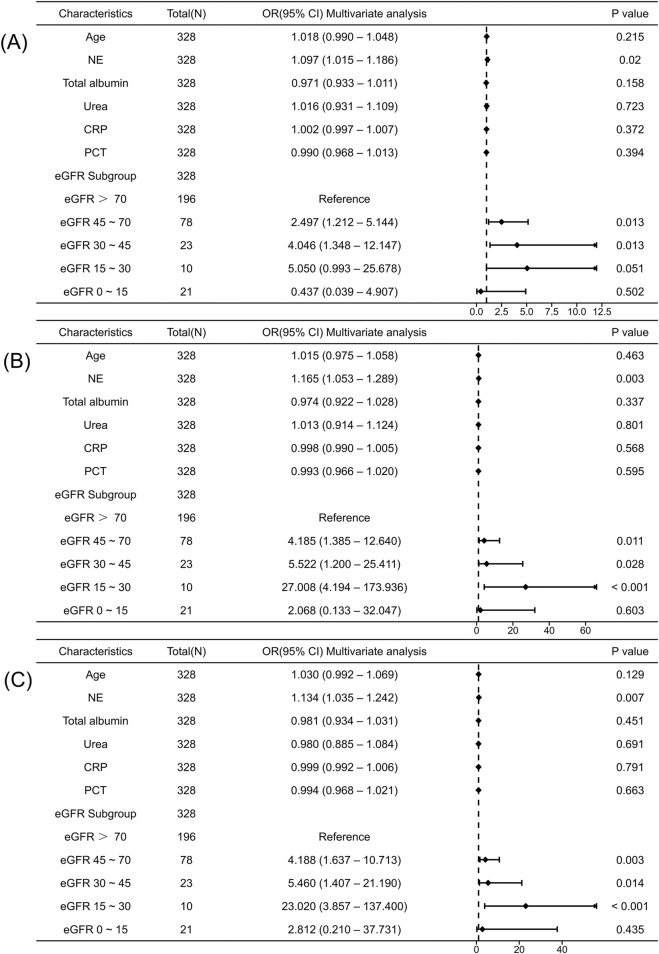
Logistics regression forest plot of renal adverse events. **(A)** Forest plot of logistic regression analysis of renal function abnormality. **(B)** Forest plot of logistic regression analysis of acute kidney injury. **(C)** Forest plot of logistic regression analysis of increased Crea.

The study group demonstrated significantly higher incidences of renal adverse events compared to the control group, including renal function abnormalities, acute kidney injury and increased Crea (all P < 0.001). Except for patients with eGFR 0–15 mL/min/1.73 m^2^, the incidence of renal adverse events in other subgroups was higher than that in the control group (Table 5). Subgroup analysis revealed a significantly higher incidence of grade 1 and grade 2 Crea elevation in the study group (P < 0.001, P = 0.023, respectively) (Table 6). FNC dosing regimens showed no significant correlations with the occurrence of the renal adverse events described above (Supplementary Table S4). Patients with elevated baseline levels of WBC, neutrophil count, Urea, CRP, or PCT prior to FNC initiation demonstrated a significantly higher incidence of renal adverse events post-treatment (P < 0.05). Furthermore, concomitant use of nephrotoxic agents was associated with a markedly elevated risk of renal function abnormalities (Supplementary Table S4). Multivariate logistic regression founded elevated neutrophil count and reduced eGFR as independent risk factors for three types of renal adverse events (Figure 4A–C).

**TABLE 5:** Analysis of ADEs of Renal function.

Project	Renal function			Acute kidney injury			Serum creatinine		
	Abnormal	Normal	P value	Abnormal	Normal	P value	Abnormal	Normal	P value
Control group (n, %)	23 (37.1)	173 (65.0)	-	6 (20.7)	190 (63.5)	-	9 (24.3)	187 (64.3)	-
Research group (n, %)	39 (62.9)	93 (35.0)	<0.001	23 (79.3)	109 (36.5)	<0.001	28 (75.7)	104 (35.7)	<0.001
Subgroup (mL/min/1.73 m^2^)	-	-	-	-	-	-	-	-	-
eGFR 45–70 (n, %)	23 (50.0)	55 (24.1)	<0.001^3^	12 (66.7)	66 (25.8)	<0.001^3^	16 (64.0)	62 (24.9)	<0.001^3^
eGFR 30–45 (n, %)	9 (28.1)	14 (7.5)	<0.001^3^	4 (40.0)	19 (9.1)	0.013^3^	5 (35.7)	18 (8.8)	0.009^3^
eGFR 15–30 (n, %)	5 (17.9)	5 (2.8)	0.005^3^	5 (45.5)	5 (2.6)	<0.001^3^	5 (35.7)	5 (2.6)	<0.001^3^
eGFR 0–15 (n, %)	2 (8.0)	19 (9.9)	1.0^3^	2 (25.0)	19 (9.1)	0.18^3^	2 (18.2)	19 (9.2)	0.29^3^
Gender (male, n, %)	44 (71.0)	175 (65.8)	0.44	22 (75.9)	197 (65.9)	0.28	26 (70.3)	193 (66.3)	0.63
Ages (Year)^1^	82.0 (73.0, 86.0)	77.0 (67.0, 84.0)	0.004	82.0 (72.0,88.0)	77.0 (67.0, 84.0)	0.040	82.0 (73.0, 87.0)	77.0 (67.0, 84.0)	0.007
BMI^2^	23.5 ± 3.0	23.43 ± 3.8	0.93	23.5 ± 2.6	23.4 ± 3.8	0.95	23.2 ± 3.1	23.5 ± 3.7	0.73
Indicators before medication	-	-	-	-	-	-	-	-	-
WBC (×10^9^/L)^1^	8.8 (5.8, 11.5)	6.3 (4.7, 8.8)	<0.001	9.5 (6.2, 12.8)	6.4 (4.8, 9.0)	<0.001	8.8 (6.0, 11.9)	6.4 (4.8, 9.0)	0.002
NE (×10^9^/L)^1^	7.3 (4.1, 9.8)	4.6 (3.3, 7.0)	<0.001	7.9 (4.8, 11.4)	4.8 (3.3, 7.4)	<0.001	7.7 (4.5, 10.6)	4.8 (3.3, 7.4)	<0.001
LY (×10^9^/L)^1^	0.70 (0.44, 0.99)	0.89 (0.58, 1.3)	0.006	0.72 (0.42, 0.96)	0.89 (0.57, 1.3)	0.013	0.70 (0.43, 0.96)	0.89 (0.57, 1.3)	0.009
Total protein (g/L)^2^	57.8 ± 9.2	60.5 ± 7.7	0.015	57.1 ± 9.8	60.3 (7.9)	0.044	57.8 ± 9.1	60.3 ± 7.9	0.082
ALB (g/L)^2^	32.5 ± 5.5	34.3 ± 5.2	0.019	32.3 ± 5.3	34.1 (5.3)	0.093	32.9 ± 5.3	34.1 ± 5.3	0.21
Urea (μmol/L)^1^	8.1 (5.9, 11.2)	6.0 (4.2, 8.7)	<0.001	10.0 (7.1, 15.9)	6.2 (4.3, 8.8)	<0.001	8.3 (5.8, 12.5)	6.2 (4.3, 8.8)	<0.001
CRP (mg/L)^1^	82.0 (34.1, 130.4)	57.0 (18.8, 101.1)	0.023	84.7 (17.6, 132.8)	58.5 (21.7, 102.4)	0.17	82.0 (28.2,132.8)	59.2 (20.6, 102.4)	0.16
PCT (ng/ml)^1^	0.18 (0.058, 1.8)	0.05 (0.05, 0.52)	<0.001	0.28 (0.075, 2.6)	0.07 (0.05, 0.52)	<0.001	0.18 (0.070, 2.2)	0.07 (0.05, 0.53)	0.003

1: median, IQR; 2: mean ± SD; 3: Compared with the control group; -: no data; BMI: body mass index; WBC: white blood cell count; NE: neutrophil count; LY: lymphocyte count; ALB: albumin; Urea: Urea nitrogen; CRP: C-reactive protein; PCT: procalcitonin.

**TABLE 6:** Analysis of the severity of elevated creatinine.

Group	Grade 1		Grade 2		Grade 3		Grade 4	
	n (%)	P value OR 95%CI	n (%)	P value OR 95%CI	n (%)	P value OR 95%CI	n (%)	P value OR 95%CI
Control group	1 (5.6)	References	2 (22.2)	References	0 (0)	-	4 (57.1)	References
Research Group	17 (94.4)	<0.001 30.9 (4.1–235.5)	7 (77.8)	0.023 6.4 (1.3–31.2)	1 (100)	-	3 (42.9)	0.69 1.4 (0.30–6.2)
eGFR Grade 1^1^	10 (55.6)	0.001 30.5 (3.8–242.9)	3 (33.3)	0.10 4.6 (0.74–28.0)	1 (100)	-	2 (28.6)	0.63 1.5 (0.27–8.5)
eGFR Grade 2^2^	4 (22.2)	0.001 42.0 (4.5–396.1)	0 (0)	-	0 (0)	-	1 (14.3)	0.40 2.6 (0.28–24.7)
eGFR Grade 3^3^	3 (16.7)	<0.001 113.4 (10.0–1,289.6)	2 (22.2)	<0.001 37.8 (4.4–325.1)	0 (0)	-	0 (0)	-
eGFR Grade 4^4^	0 (0)	-	2 (22.2)	0.026 10.0 (1.3–74.7)	0 (0)	-	0 (0)	-

no data; 1: eGFR, 45–70 mL/min/1.73 m^2^; 2: eGFR, 30–45 mL/min/1.73 m^2^; 3: eGFR, 15–30 mL/min/1.73 m^2^; 4: eGFR <15 mL/min/1.73 m^2^; OR: odds ratio; 95% CI: 95% confidence interval.

### 3.5 Age-stratified analysis

Age-stratified analyses revealed that patients aged <60 in the study group exhibited numerically higher incidences of total ADEs, hepatic function abnormalities, and renal parameter deviations compared to controls, but these differences did not reach statistical significance (Table 7; Supplementary Table S7). Notably, patients aged ≥80 in the study group demonstrated significantly elevated GGT levels compared to controls (P = 0.027). The populations aged ≥60 in the study group showed significantly increased rates of renal function abnormalities and serum Crea elevation (all P < 0.05). This trend was most pronounced for acute kidney injury (AKI) incidence in the population aged 60–80 in the study group (P < 0.05).

**TABLE 7:** Age stratified analysis.

Subjects	Aged <60			Aged 60–80			Aged ≥80		
	G1^1^ n (%)	G2^2^ n (%)	P value OR 95%CI	G1^1^ n (%)	G2^2^ n (%)	P value OR 95%CI	G1^1^ n (%)	G2^2^ n (%)	P value OR 95%CI
Total	33 (100)	10 (100)	References	90 (100)	50 (100)	References	73 (100)	72 (100)	References
Overall ADEs	8 (24.2)	4 (40.0)	0.34 2.1 (0.47–9.3)	46 (51.1)	27 (54.0)	0.74 1.1 (0.56–2.2)	38 (52.1)	47 (65.3)	0.11 1.7 (0.89–3.4)
Renal function^3^	3 (9.1)	2 (20.0)	0.36 2.5 (0.35–17.6)	9 (10.0)	13 (26.0)	0.016 3.2 (1.2–8.1)	11 (15.1)	24 (33.3)	0.012 2.8 (1.3–6.3)
Kidney injury^4^	0 (0)	2 (20.0)	-	1 (1.1)	9 (18.0)	0.006 19.5 (2.4–159.4)	5 (6.8)	12 (16.7)	0.074 2.7 (0.91–8.2)
Elevated creatinine	0 (0)	2 (20.0)	-	2 (2.2)	10 (20.0)	0.003 11.0 (2.3–52.5)	7 (9.6)	16 (22.2)	0.042 2.7 (1.0–7.0)

no data; 1: Control group; 2: Research group; 3: Abnormal renal function indicators; 4: Acute kidney injury; OR: odds ratio; 95% CI: 95% confidence interval.

### 3.6 Severity stratification of COVID-19 analysis

Compared with patients with mild to moderate COVID-19, the incidence rates of total ADEs, renal function abnormalities, AKI, elevated serum creatinine, hepatic function abnormalities, increased transaminase and elevated GGT in severe and critical COVID-19 patients were significantly higher (all P < 0.001) (Supplementary Table S8). Stratified analysis was performed according to COVID-19 severity to explore its influence on FNC-associated ADEs. Among patients with mild and moderate COVID-19, no significant differences were observed between the control and study groups in the above-mentioned ADEs. For severe and critical COVID-19 cases, the study group exhibited markedly higher incidences of renal function abnormalities, AKI and elevated serum creatinine relative to the control group (all P < 0.05) (Supplementary Table S9).

### 3.7 Establishment of the nomogram model for predicting renal adverse events in patients taking FNC

**FIGURE 5:**
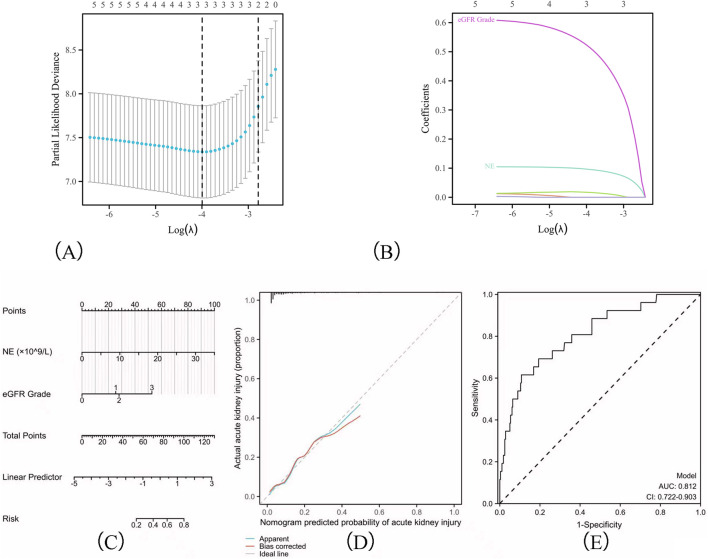
Prediction model for azvudine-associated acute kidney injury (AKI). **(A)** LASSO coefficient profiles of the 6 features for AKI. **(B)** Tuning parameter (λ) selection in the LASSO model. **(C)** Nomogram of AKI. **(D)** Calibration curve of nomogram in apparent and bias corrected of AKI. **(E)** ROC curve of nomogram of AKI.

Considering the high frequency of AKI (8.8%) in our retrospective analysis and its significant correlation with eGFR grade, we attempted to establish a nomogram model for predicting FNC-associated AKI. Variables with P < 0.05 in the baseline analysis of AKI were selected for inclusion in the univariate logistic regression analysis. The univariate analysis indicated that NE, lymphocyte (LY), Urea and eGFR grades 1–3 were significantly associated with AKI. A total of 26 AKI events were included in the multivariable logistic regression analysis. In the multivariate logistic regression analysis, these indicators showed significant differences, including: NE (P = 0.0007; Odds Ratio (OR) = 1.18, 95% confidence interval (CI): 1.07–1.31), eGFR Grade 1 (P = 0.005; OR = 4.52,95% CI: 1.57–12.99), eGFR Grade 2 (P = 0.021; OR = 5.25, 95% CI: 1.28–21.45), and eGFR Grade 3 (P = 0.0003; OR = 22.80, 95% CI: 1.28–21.45). The VIF values of the above indicators are 1.013, 1.39, 1.29, and 1.18 respectively, indicating that there is no serious multicollinearity among these indicators. Both NE and eGFR grades have non-zero coefficients in the LASSO regression model. Based on the results of the multivariate analysis and LASSO regression, a nomogram model was developed to predict AKI in patients taking FNC (Figure 5). Calibration curves for the nomogram demonstrated good agreement between apparent and bias-corrected values. The Hosmer-Lemeshow goodness-of-fit test was adopted to assess model calibration, with P = 0.6855 indicating satisfactory fitting performance. The likelihood ratio test was used for overall model verification, and P < 0.001 suggested statistical significance of the established model. ROC curve analysis showed that the AUC and 95% confidence interval of the model was 0.812 (0.722–0.903), with a sensitivity of 0.61, and specificity of 0.89.

## 4 Discussion

In the current study, COVID-19 patients with renal impairment undergoing FNC treatment demonstrated a significantly higher incidence of ADEs, particularly renal-related ADEs. Considering that FNC is mainly excreted via the kidney, systemic accumulation of FNC and its metabolites may occur in patients with renal impairment, which elevates the risk of drug-associated toxicity. Yu et al. and Rao et al., 2025 reported that FNC did not elevate the overall incidence of ADEs in patients with kidney diseases (Yu et al., 2025). The findings of the present study contradicted their results, primarily due to differences in grouping design among the three studies. Yu et al. enrolled patients without antiviral treatment as controls, while Rao et al., 2025 took patients receiving Paxlovid as the control group. In this study, patients treated with FNC and having an eGFR exceeding 70 mL/min/1.73 m^2^ were defined as controls. Additionally, they documented higher incidence rates of certain individual adverse events in the FNC group, including elevated ALT, increased ALP and thrombocytopenia.

Stratified analysis based on COVID-19 severity revealed that severe and critical COVID-19 patients complicated with renal injury were more susceptible to renal-related ADEs after FNC administration. Although mild and moderate COVID-19 patients with concomitant renal injury presented a relatively high incidence of renal-related ADEs following FNC treatment, no statistically significant difference was found compared with the control group. Severe SARS-CoV-2 infection aggravated renal injury (Pakhchanian et al., 2021). Renal damage further accumulated drugs predominantly excreted via the kidney. The superimposed effects of these two factors might elevate the risk of renal injury in severe pneumonia patients receiving FNC.

Logistic regression analysis identified baseline neutrophil count as an independent factor for FNC-related ADEs, especially renal ADEs, highlighting systemic inflammation’s role in drug toxicity. There is evidence suggesting that chronic inflammation worsens drug-induced organ damage (Furman et al., 2019). Pro-inflammatory cytokines suppress cytochrome P450 enzyme activity, amplifying drug exposure (Wang et al., 2022). Immune hyperactivation in inflammatory states may promote drug-antigen recognition, triggering attacks on renal and hepatic tissues, accelerating organ injury (Karki and Kanneganti, 2022). Logistic regression analysis further identified advanced age as an independent risk factor for FNC-associated ADEs. Age-stratified analyses revealed significantly higher rates of renal function abnormalities among COVID-19 patients aged ≥60 years in the study group. Notably, the incidence of AKI after FNC treatment was markedly elevated in patients aged 60–80 years (P < 0.05). Age-related progressive decline in hepatic and renal metabolic function is accompanied by reduced drug clearance and heightened susceptibility to drug-induced toxicity (Wu et al., 2023). Polypharmacy increases risk of adverse events from complex drug interactions (Ornstein et al., 2013). Tan reported that 60% of ADEs in patients ≥65 years originated from interactions involving anticoagulants, hypoglycemics, or opioids (Tan et al., 2018).

Subgroup analyses demonstrated a graded escalation in ADE risk with declining eGFR, peaking in patients with moderate-to-severe renal impairment (eGFR 15–45 mL/min/1.73 m^2^). Surprisingly, no significant increase in renal toxicity was observed in patients with severe renal impairment (eGFR <15 mL/min/1.73 m^2^). This paradox may stem from several factors, including dose adjustment, dialysis treatment, and the small sample size. Overall, clinicians reduced the dose of FNC in 14 patients, including 7 patients with eGFR ranging from 0 to 30 mL/min/1.73 m^2^, thereby reducing systemic drug accumulation. Additionally, 52% of patients with eGFR <15 mL/min/1.73 m^2^ received peritoneal dialysis or hemodialysis. Subgroup analysis revealed no significant difference in ADEs between the dialysis group and the non-dialysis group (3.5% vs. 6.3%, P > 0.05), suggesting that dialysis may enhance the clearance of FNC. Published studies reported that azvudine treatment did not increase adverse hepatic and renal events in hemodialysis patients compared with conventional therapy (Shang et al., 2023). Finally, only 21 patients with eGFR <15 mL/min/1.73 m^2^ were included. In the future, it is hoped that more samples will be available for verification.

There are currently no studies on the monitoring of plasma FNC concentrations in patients with renal impairment. Considering that azvudine is mainly excreted via the kidneys in its prototype form, which accounts for over 70% of the total excretion, dose reduction of azvudine is required for patients with renal impairment complicated with COVID-19 infection. It is recommended to reduce the daily dose to 3 mg in patients with eGFR of 30–60 mL/min/1.73 m^2^, and to 1–2 mg daily in those with eGFR of 0–30 mL/min/1.73 m^2^. Patients receiving dialysis treatment may maintain the daily dose of 5 mg.

Multivariate logistic regression identified elevated neutrophil count and reduced eGFR as independent risk factors for renal ADEs, especially AKI. The eGFR is a major risk factor for serious adverse drug reactions. The risk of acute kidney injury was 2.2% higher for each 1 mL/min/1.73m2 lower baseline eGFR (Laville et al., 2024). Renal injury triggers the release of damage-associated molecular patterns (DAMPs), which activate innate immune responses during viral infection (Tian et al., 2025). DAMPs initiate cascades of pro-inflammatory mediators, chemokines, and reactive oxygen species, ultimately driving renal tubular apoptosis and fibrotic remodeling (Roh and Sohn, 2018; Pavlakou et al., 2017; Sato and Yanagita, 2018). To better facilitate clinical treatment, this study attempted to establish a prediction model for FNC-associated AKI based on neutrophil count and eGFR levels. ROC curve analysis yielded that the AUC and 95% confidence interval of the model was 0.812 (0.722–0.903), suggesting predictive potential. Nevertheless, this model was constructed solely using retrospective data, and external validation was not performed due to insufficient sample size. Further validation with expanded datasets is required prior to clinical application.

Although no significant intergroup differences were observed in the overall incidence of hepatic ADEs, subgroup analyses revealed markedly higher rates in patients with eGFR 30–45 mL/min/1.73 m^2^, particularly for Grade 2 transaminase elevation and Grade 1 GGT elevation. Declined renal function may cause uremic toxin accumulation, worsening liver burden through hepatorenal crosstalk, oxidative stress, and inflammation (Arakawa and Kato, 2023). Logistic regression identified elevated CRP levels and concomitant use of hepatotoxic agents as independent risk factors for hepatic ADEs. Prior research confirms that severe COVID-19 is linked to liver injury, hepatic failure, and increased TNF-α. In chronic inflammation, TNF-α activates Kupffer cells and causes oxidative stress in liver cells. This inflammation reduces metabolic enzyme activity, impairing drug detoxification and raising hepatotoxicity risks (Lucke et al., 2023; Huang et al., 2020). Polypharmacy with hepatotoxic agents worsens liver injury, likely via drug interactions causing metabolic overload or mitochondrial dysfunction in hepatocytes (Chen et al., 2023).

## 5 Limitations

Admittedly, this study has several limitations that have important implications for further research. First, the current retrospective design may introduce biases, particularly due to inconsistent monitoring of hepatic/renal function and hematologic parameters during FNC treatment, which may explain the lack of observed correlations between treatment duration and adverse events. Second, this study is limited by its small sample size. Subgroups with eGFR 15–30 mL/min/1.73 m^2^ and <15 mL/min/1.73 m^2^ were underrepresented, which may have masked FNC-associated risks in these populations. Third, renal assessments relied primarily on serum Crea and BUN, as sensitive markers (e.g., urinary protein, cystatin C) were rarely monitored during clinical care. Fourth, diverse underlying diseases and concurrent medications may trigger adverse events and introduce bias to the research findings. Due to the scattered and extensive data, these factors could not be individually identified in this study. Furthermore, the ADEs prediction model analyzed only abnormalities in renal function, and we did not collect an additional cohort for external validation. The prediction model lacked external validation, and its accuracy and generalizability remained to be further investigated. We hope to collect more patients for validation in the future.

## 6 Conclusion

In summary, this study highlights the heightened risk of FNC-associated ADEs in COVID-19 patients with renal impairment, with elevated neutrophil count and advanced age identified as independent risk factors. The lower the eGFR, the more likely the patients had abnormal hepatic or kidney function after using FNC. These findings underscore the necessity for vigilant safety monitoring and individualized dose adjustments of FNC treatment in populations wiht renal dysfunction. Future large-scale clinical studies are imperative to validate these observations and optimize risk-stratified therapeutic strategies for SARS-CoV-2 infection in vulnerable cohorts.

## Data Availability

The raw data supporting the conclusions of this article will be made available by the authors, without undue reservation.
