# Supplementary material for: Safety profile of azvudine in COVID-19 patients with renal impairment: a retrospective analysis
Source: Front Pharmacol. 2026 Jun 29;17:1830085. doi: 10.3389/fphar.2026.1830085 (PMC13357969; doi:10.3389/fphar.2026.1830085)
Supplement: Supplementary file 1 [file Table1.docx]

Supplementary Table 1 Screening criteria of adverse events associated with azvudine

| Type of Adverse Events | Criteria of Adverse Events |
| --- | --- |
| Drug-associated liver function abnormalities | post-treatment elevation of liver enzymes beyond upper limit of normal (ULN), including ALT, AST, ALP, GGT, TBIL, DBIL, and IBIL. For patients with pre-existing liver dysfunction, progressive liver enzyme elevation post-treatment was required. |
| Drug-induced liver injury | 1. ALT ≥5×ULN; 2. ALP ≥2×ULN (with concurrent GGT elevation, excluding bone disease-related ALP elevation); 3. ALT ≥3×ULN + TBIL ≥2×ULN; 4. post-treatment doubling of liver enzymes from baseline in patients with pre-existing abnormalities, unexplained by underlying liver disease |
| Drug-associated renal function abnormalities | post-treatment elevation of serum creatinine (Crea) or blood urea nitrogen (Urea) > 1×ULN. For patients with pre-existing renal dysfunction, progressive elevation of Crea or Urea post-treatment was required. |
| Acute kidney injury | Crea increase >26.5 µmol/L from baseline, or Crea ≥1.5× baseline level |
| Diarrhea | New-onset loose stools documented as "diarrhea" post-azvudine initiation, without prior laxative use (including herbal agents) or history of enteritis. |
| Nausea/Vomiting | events temporally linked to azvudine administration without alternative explanations. |
| hematologic adverse events | Anemia (hemoglobin level <10 g/dL), leukopenia (white blood cell count <4500 cells/μL), or thrombocytopenia (plateletcount <150 × 10^3^/μL) with levels below patient’s baseline and in the absence of bleeding or myelosuppressive therapies. |
| hypersensitivity reactions | Rash, including hives, nonhives rashes, and red man syndrome, temporally associated with azvudine administration with resolution on azvudine discontinuation. |
| neurologic toxicities | Altered mental status, peripheral neuropathy, or seizures in the absence of preexisting neurologic conditions, substance-related toxic effects, or infectious syndromes. |

Note：ALT: Alanine transaminase; AST: Aspartate aminotransferase; ALP: Alkaline phosphatase; GGT: γ-glutamyl transpeptidase; TBIL: Total bilirubin; DBIL: Direct Bilirubin, IBIL: Indirect bilirubin.

Supplementary Table 2 Criteria for evaluating the severity of toxicity according to the type of ADEs

| Type of Adverse Events | Grade | Definiton |
| --- | --- | --- |
| transaminase elevation | Grade 0 | No elevation beyond ULN, or ≤1× baseline in patients with pre-existing abnormalities; |
|  | Grade 1 | 1×ULN < ALT or AST ≤3×ULN; or <1× baseline increase in abnormal baseline cases; |
|  | Grade 2 | 3×ULN < ALT or AST <5×ULN, or 1–3× baseline increase; |
|  | Grade 3 | ALT or AST ≥5×ULN, or >3× baseline increase. |
| GGT elevation | Grade 0 | GGT ≤ ULN, or ≤1× baseline in pre-existing abnormalities; |
|  | Grade 1 | 1×ULN < GGT ≤3×ULN, or <1× baseline increase; |
|  | Grade 2 | 3×ULN < GGT ≤5×ULN, or 1–3× baseline increase; |
|  | Grade 3 | GGT >5×ULN, or >3× baseline increase. |
| Crea elevation | Grade 0 | ≤ULN, or no increase from abnormal baseline; |
|  | Grade 1 | >1×ULN but <1.5× baseline; |
|  | Grade 2 | > ULN and 1.5~2× baseline |
|  | Grade 3 | > ULN and 2~3× baseline; |
|  | Grade 4 | > ULN and >3× baseline or requiring renal replacement therapy (RRT). |

Note：ULN: upper limit of normal; ALT: Alanine transaminase; AST: Aspartate aminotransferase; ALP: Alkaline phosphatase; GGT: γ-glutamyl transpeptidase; TBIL: Total bilirubin; DBIL: Direct Bilirubin; IBIL: Indirect bilirubin; Crea: serum creatinine.

Supplementary Table 3 The influence of FNC administration on liver function

| Project | Hepatic parameter | | | Transaminase elevation | | | GGT elevation | | |
| --- | --- | --- | --- | --- | --- | --- | --- | --- | --- |
|  | Abnormal | Normal | P value | Abnormal | Normal | P value | Abnormal | Normal | P value |
| FNC dosage (mg)^1^ | 5.0 (2.0, 5.0) | 5.0 (2.0, 5.0) | 0.54 | 5.0 (2.0, 5.0) | 5.0 (2.0, 5.0) | 0.93 | 5.0 (3.0, 5.0) | 5.0 (2.0, 5.0) | 0.48 |
| FNC treatment duration (d)^1^ | 7.0 (1.0, 14.0) | 7.0 (1.0, 17.0) | 0.19 | 7.0 (1.0, 14.0) | 7.0 (1.0, 17.0) | 0.13 | 7.0 (1.0, 14.0) | 7.0 (1.0, 17.0) | 0.42 |
| FNC cumulative dose (mg)^1^ | 35.0 (5.0, 70.0) | 35.0 (5.0, 85.0) | 0.34 | 35.0 (5.0, 70.0) | 35.0 (5.0, 85.0) | 0.21 | 35.0 (5.0, 70.0) | 35.0 (5.0, 85.0) | 0.65 |

Note: FNC: Azvudine; 1: median , IQR.

Supplementary Table 4 The influence of FNC administration and combined medication on renal function

| Project | Renal function | | | Acute kidney injury | | | Serum creatinine | | |
| --- | --- | --- | --- | --- | --- | --- | --- | --- | --- |
|  | Abnormal | Normal | P value | Abnormal | Normal | P value | Abnormal | Normal | P value |
| FNC dosage (mg)^1^ | 5.0 (2.0, 5.0) | 5.0 (2.0, 5.0) | 0.52 | 5.0 (5.0, 5.0) | 5.0 (2.0, 5.0) | 0.20 | 5.0 (2.0, 5.0) | 5.0 (2.0, 5.0) | 0.54 |
| FNC treatment duration (d)^1^ | 1. (2.0, 17.0) | 7.0 (1.0, 14.0) | 0.20 | 7.0 (2.0, 14.0) | 7.0 (1.0, 17.0) | 0.78 | 7.0 (2.0, 14.0) | 7.0 (1.0, 17.0) | 0.58 |
| FNC cumulative dose (mg)^1^ | 35.0 (10.0, 85.0) | 35.0 (5.0, 70.0) | 0.27 | 35.0 (10.0, 70.0) | 35.0 (5.0, 85.0) | 0.53 | 35.0 (10.0, 70.0) | 35.0 (5.0, 85.0) | 0.65 |
| Combined drugs with the commonly incidence of abnormal renel function (n, %) | 19 (30.6) | 46 (17.3) | 0.018 | 8 (27.6) | 57 (19.1) | 0.27 | 11 (29.7) | 54 (18.6) | 0.11 |
| Combined the use of ACEI or ARB (n, %) | 7 (11.3) | 20 (7.5) | 0.33 | 5 (17.2) | 22 (7.4) | 0.064 | 6 (16.2) | 21 (7.2) | 0.10 |

Note: FNC: Azvudine; 1: median , IQR; ACEI: Angiotensin-Converting Enzyme Inhibitors; ARB: Angiotensin Receptor Blockers.

Supplementary Table 5 Analysis of the severity of elevated transaminase

| Group | Grade 1 | | Grade 2 | | Grade 3 | |
| --- | --- | --- | --- | --- | --- | --- |
|  | n  (%) | P value  OR 95%CI | n  (%) | P value  OR 95%CI | n  (%) | P value  OR 95%CI |
| Control group | 36  (52.9) | Reference | 19  (59.4) | Reference | 3  (42.9) | Reference |
| Research  Group | 32  (47.1) | 0.16  1.5 (0.85-2.6) | 13  (40.6) | 0.74  1.1 (0.53-2.4) | 4  (57.1) | 0.30  2.2 (0.48-10.1) |
| eGFR  Grade 1^1^ | 23  (33.8) | 0.062  1.8 (0.97-3.3) | 4  (12.5) | 0.36  0.59 (0.19-1.8) | 2  (28.6) | 0.50  1.9 (0.30-11.6) |
| eGFR  Grade 2^2^ | 7  (10.3) | 0.085  2.4 (0.88-6.7) | 5  (15.6) | 0.044  3.3 (1.0-10.5) | 0  (0) | - |
| eGFR  Grade 3^3^ | 1  (1.5) | 0.58  0.55 (0.065-4.6) | 1  (3.1) | 0.97  1.0 (0.12-8.9) | 1  (14.3) | 0.12  6.6 (0.60-71.5) |
| eGFR  Grade 4^4^ | 1  (1.5) | 0.17  0.24 (0.031-1.9) | 3  (9.4) | 0.65  1.4 (0.36-5.1) | 1  (14.3) | 0.37  2.9 (0.28-29.3) |

Note: -: No data; 1: eGFR 45~70 mL/min/1.73 m²; 2: eGFR 30~45 mL/min/1.73 m²; 3: eGFR 15~30 mL/min/1.73 m²; 4: eGFR <15 mL/min/1.73 m².

Supplementary Table 6 Analysis of the severity of elevated GGT

| Group | Grade 1 | | Grade 2 | | Grade 3 | |
| --- | --- | --- | --- | --- | --- | --- |
|  | n  (%) | P value  OR 95%CI | n  (%) | P value  OR 95%CI | n  (%) | P value  OR 95%CI |
| Control group | 24  (50.0) | Reference | 4  (40.0) | Reference | 3  (60.0) | Reference |
| Research Group | 24  (50.0) | 0.11  1.7 (0.89-3.1) | 6  (60.0) | 0.17  2.5 (0.68-9.0) | 2  (40.0) | 0.92  1.1 (0.18-6.7) |
| eGFR  Grade 1^1^ | 12  (25.0) | 0.36  1.4 (0.67-3.0) | 6  (60.0) | 0.029  4.3 (1.2-15.7) | 2  (40.0) | 0.49  1.9 (0.31-11.6) |
| eGFR  Grade 2^2^ | 9  (18.8) | 0.002  4.4 (1.7-11.3) | 0  (0) | - | 0  (0) | - |
| eGFR  Grade 3^3^ | 1  (2.1) | 0.80  0.76 (0.093-6.3) | 0  (0) | - | 0  (0) | - |
| eGFR  Grade 4^4^ | 2  (4.2) | 0.68  0.72 (0.16-3.3) | 0  (0) | - | 0  (0) | - |

Note: -: No data; 1: eGFR 45~70 mL/min/1.73 m²; 2: eGFR 30~45 mL/min/1.73 m²; 3: eGFR 15~30 mL/min/1.73 m²; 4: eGFR <15 mL/min/1.73 m².

Supplementary Table 7 Age-stratified analysis of the impact on ADEs related to liver function

| Group | Aged ＜60 | | | Aged 60 ~ 80 | | | Aged ≥ 80 | | |
| --- | --- | --- | --- | --- | --- | --- | --- | --- | --- |
|  | G1^1^  n (%) | G2^2^  n (%) | P value  OR 95%CI | G1^1^  n (%) | G2^2^  n (%) | P value  OR 95%CI | G1^1^  n (%) | G2^2^  n (%) | P value  OR 95%CI |
| Total | 33  (100) | 10  (100) | Reference | 90  (100) | 50  (100) | Reference | 73  (100) | 72  (100) | Reference |
| Hepatic parameter^3^ | 5  (15.2) | 4  (40.0) | 0.10  3.7 (0.77-18.2) | 33  (36.7) | 20  (40.0) | 0.70  1.2 (0.57-2.3) | 27  (37.0) | 32  (44.4) | 0.36  1.4 (0.70-2.7) |
| Transaminase elevation | 5  (15.2) | 3  (30.0) | 0.30  2.4 (0.46-12.5) | 29  (32.2) | 19  (38.0) | 0.49  1.3 (0.63-2.7) | 24  (32.9) | 27  (37.5) | 0.56  1.2 (0.62-2.4) |
| GGT elevation | 4  (12.1) | 2  (20.0) | 0.53  1.8 (0.28-11.8) | 20  (22.2) | 13  (26.0) | 0.61  1.2 (0.55-2.8) | 7  (9.6) | 17  (23.6) | 0.027  2.9 (1.1-7.5) |

Note: 1: Control group; 2: Research group; 3: Hepatic parameter abnormal.

Supplementary Table 8 The impact of the severity of COVID-19 on ADEs

| Group | Mild to moderate COVID-19 | | Severe/critical COVID-19 | | P value  OR 95%CI |
| --- | --- | --- | --- | --- | --- |
|  | Normal | Abnormal | Normal | Abnormal |  |
| Overall ADEs  n (%) | 119  (61.3) | 75  (38.7) | 39  (29.1) | 95  (70.9) | ＜0.001  3.87 (2.41-6.19) |
| Abnormal renal function indicators  n (%) | 180  (92.8) | 14  (7.2) | 86  (64.2) | 48  (35.8) | ＜0.001  7.18 (3.75-13.72) |
| Acute kidney injury  n (%) | 191  (98.5) | 3  (1.5) | 108  (80.6) | 26  (19.4) | ＜0.001  15.33 (4.53-51.82) |
| Elevated creatinine  n (%) | 188  (96.9) | 6  (3.1) | 103  (76.9) | 31  (23.1) | ＜0.001  9.43 (3.81-23.35) |
| Hepatic parameter abnorma  n (%) | 147  (75.8) | 47  (24.2) | 60  (44.8) | 74  (55.2) | ＜0.001  3.86 (2.40-6.19) |
| Transaminase elevation  n (%) | 155  (79.9) | 39  (20.1) | 66  (49.3) | 68  (50.7) | ＜0.001  4.09 (2.51-6.67) |
| GGT elevation  n (%) | 173  (89.2) | 21  (10.8) | 92  (68.7) | 42  (31.3) | ＜0.001  3.76 (2.10-6.73) |

Supplementary Table 9 Severity stratification of COVID-19 analysis of the impact on ADEs

| Group | Mild to moderate COVID-19 | | | Severe/critical COVID-19 | | |
| --- | --- | --- | --- | --- | --- | --- |
|  | G1^1^  n (%) | G2^2^  n (%) | P value  OR 95%CI | G1^1^  n (%) | G2^2^  n (%) | P value  OR 95%CI |
| Total | 129  (100) | 65  (100) | Reference | 67  (100) | 67  (100) | Reference |
| Overall ADEs | 48  (37.2) | 27  (41.5) | 0.56  1.12 (0.78-1.61) | 44  (65.7) | 51  (76.1) | 0.18  1.16 (0.93-1.44) |
| Renal function^3^ | 6  (4.7) | 8  (12.3) | 0.075  2.65 (0.96-7.31) | 17  (25.4) | 31  (46.3) | 0.012  1.82 (1.12-2.96) |
| Kidney injury^4^ | 0  (0.0) | 3  (4.6) | - | 6  (9.0) | 20  (29.9) | 0.002  3.33 (1.43-7.78) |
| Elevated creatinine | 2  (1.6) | 4  (6.2) | 0.098  3.97 (0.75-21.11) | 7  (10.4) | 24  (35.8) | ＜0.001  3.43 (1.59-7.41) |
| Hepatic parameter^5^ | 30  (23.3) | 17  (26.2) | 0.66  1.12 (0.67-1.88) | 35  (52.2) | 39  (58.2) | 0.49  1.11 (0.82-1.51) |
| Transaminase elevation | 25  (19.4) | 14  (21.5) | 0.72  1.11 (0.62-1.99) | 33  (49.3) | 35  (52.2) | 0.73  1.06 (0.76-1.48) |
| GGT elevation | 12  (9.3) | 9  (13.8) | 0.34  1.49 (0.66-3.35) | 19  (28.4) | 23  (34.3) | 0.46  1.21 (0.73-2.01) |

Note: 1: Control group; 2: Research group; 3: Abnormal renal function indicators; 4: Acute kidney injury; 5: Hepatic parameter abnormal.
